# Microbial lipid production from crude glycerol and hemicellulosic hydrolysate with oleaginous yeasts

**DOI:** 10.1186/s13068-021-01916-y

**Published:** 2021-03-12

**Authors:** Mikolaj Chmielarz, Johanna Blomqvist, Sabine Sampels, Mats Sandgren, Volkmar Passoth

**Affiliations:** Department of Molecular Sciences, Swedish University of Agricultural Sciences, Uppsala, Sweden

**Keywords:** *Oleaginous yeast*, *Hemicelluloses*, *Crude glycerol*, *Lipids*, *R. torouloides*

## Abstract

**Background:**

Crude glycerol (CG) and hemicellulose hydrolysate (HH) are low—value side-products of biodiesel transesterification and pulp—and paper industry or lignocellulosic ethanol production, respectively, which can be converted to microbial lipids by oleaginous yeasts. This study aimed to test the ability of oleaginous yeasts to utilise CG and HH and mixtures of them as carbon source.

**Results:**

Eleven out of 27 tested strains of oleaginous yeast species were able to grow in plate tests on CG as sole carbon source. Among them, only one ascomycetous strain, belonging to *Lipomyces starkeyi*, was identified, the other 10 strains were *Rhodotorula* spec. When yeasts were cultivated in mixed CG/ HH medium, we observed an activation of glycerol conversion in the *Rhodotorula* strains, but not in *L. starkeyi*. Two strains—*Rhodotorula toruloides* CBS 14 and *Rhodotorula glutinis* CBS 3044 were further tested in controlled fermentations in bioreactors in different mixtures of CG and HH. The highest measured average biomass and lipid concentration were achieved with *R. toruloides* in 10% HH medium mixed with 55 g/L CG—19.4 g/L and 10.6 g/L, respectively, with a lipid yield of 0.25 g lipids per consumed g of carbon source. Fatty acid composition was similar to other *R. toruloides* strains and comparable to that of vegetable oils.

**Conclusions:**

There were big strain differences in the ability to convert CG to lipids, as only few of the tested strains were able to grow. Lipid production rates and yields showed that mixing GC and HH have a stimulating effect on lipid accumulation in *R. toruloides* and *R. glutinis* resulting in shortened fermentation time to reach maximum lipid concentration, which provides a new perspective on converting these low-value compounds to microbial lipids.

**Supplementary Information:**

The online version contains supplementary material available at 10.1186/s13068-021-01916-y.

## Background

There is increased need for recycling of waste products from food, wood industry and agriculture in recent years. Vast quantities of organic residues are a challenge that is being addressed by converting waste and low purity sugars into higher quality products [1].

Crude glycerol (CG) is a side product of biodiesel production, which is released during transesterification of vegetable oils. CG is a highly problematic side product; it contains methanol, soap and ash. Industrial application of CG requires extensive purification, making its application quite expensive [2–5]. In recent years there have been developments to utilse glycerol and convert it to useable biomass and for example turn it into microbial fuel cells [6], or to convert glycerol into valuable fatty acids using *Yarrowia lipolytica* [7]. It was also tested as an additive with other waste substrates to make microbial oil and other valuable products [8–13].

Hemicellulose is, besides of cellulose and lignin, one of the major polymers of the plant cell wall. It is a heteropolysaccharide, which composition differs between different groups of plants. Hardwood hemicellulose consists mainly of xylans, while softwood is mainly built of glucomannans. Hemicellulose of lignocellulosic materials of monocoyledons like wheat straw consist of a xylan backbone with side chains of arabinose and glucuronic acid [14, 15]. In pulp and paper industry, hemicellulose is a side product and used for steam generation. However, it is rather inefficient for this application, due to its low heating value (13.5 MJ/ kg) [5]. In lignocellulose-based bioethanol production, separating hemicellulose from the cellulose fraction can be advantageous, since conversion of the pentoses, which are the major sugar monomers of hemicellulose to ethanol is still a challenge [7, 8]. When lignocellulose is thermochemically pretreated by acid hydrolysis, hemicellulose is solubilised and hydrolysed, resulting in the release of monosaccharides [16]. Since the major parts of cellulose and lignin are still in the solid phase, it is easy to separate the hemicellulose hydrolysate from the other components of lignocellulose hydrolysate. Hemicellulose hydrolysates contain, in addition to released sugars, a variety of inhibitors such as furaldehydes, weak organic acids and phenolic compounds [17], and frequently it is necessary to remove those inhibitors or to dilute hemicellulose hydrolysate to enable bioconversion of hemicellulose hydrolysates [13, 18, 19].

Many oleaginous yeasts, i.e., yeasts that can accumulate more than 20% of their biomass as lipids, can convert both glycerol as well as sugars and organic acids derived from hemicellulose to oil. This oil has a similar composition as some vegetable oils [20]. Production of vegetable oil can have considerable greenhouse gas potential [21] and thus, replacement of vegetable oil by yeast oil may result in more sustainable biodiesel [22, 23] or animal feed production [24]. However, production costs of microbial oil are still too high to be competitive with oil from fossil resources or vegetable oils [25, 26]. Therefore, it is important to explore the conversion of low-value side products such as lignocellulose hydrolysate, hemicellulose hydrolysate, or crude glycerol to microbial oils. Another approach to improve cost efficiency of microbial lipid production is to identify strains with high productivity when converting the mentioned residual products to lipids and to understand their physiology, to identify targets for strain optimisation [27, 28].

Converting a mixture of CG and HH to yeast oil may provide a possibility of simultaneously converting these side streams to a high-value products. At the same time, inhibitors present in only one of these side streams might be diluted and thus less inhibitory. It was our aim to get a survey about the diversity of strains when converting CG and HH to lipids. We also intended to investigate effects of mixing these two problematic substrates. We tested several of strains belonging to the genera *Lipomyces* (ascomyceteous oleaginous yeasts) and *Rhodotorula* (basidiomycetous oleaginous yeasts) for their ability to grow and synthetise lipids on a mixture of CG and HH.

## Results

### Crude glycerol and hemicellulose growth tests

The ability of 27 yeast strains to utilise CG as sole carbon source and their resistance against inhibitors in CG was tested on solid media. A droplet of undiluted crude glycerol or a filter soaked with CG was placed at the centre of YNB plates inoculated with the tested strains. Out of the 27 tested strains, 11 strains showed visible growth close to the droplet/filter and thus were able to tolerate CG, and to utilise it as carbon source (experiments were performed in duplicates). Those strains were: *Rhodotorula glutinis* CBS 2203, *R. glutinis* CBS 2889, *R. glutinis* CBS 2890, *R. glutinis* CBS 3044, *R. glutinis* CBS 5182, *R. glutinis* CBS 7538, *Rhodotorula minuta* CBS 8013, *Rhodotorula mucolaginosa* CFSQE 63, *Rhodotorula babjevae* CBS 7809, *Rhodotorula toruloides* CBS 14 and *Lipomyces starkeyi* CBS 7786. Interestingly, only one ascomycetous strain (*L. starkeyi* 7786) showed substantial growth on the plates with crude glycerol. These strains were also tested in liquid shake flask cultures with 40% hemicellulose hydrolysate as sole carbon source, and in cultures with different concentrations of crude glycerol (up to 120 g/l) as sole carbon source. After 96 h of cultivation almost all red yeast strains grew well in hemicellulose hydrolysate (OD 20–40) and in 120 g/L CG (OD > 40). *R. minuta* CBS 8013 grew slower than other *Rhodotorula* strains in CG concentrations higher than 60 g/L. *L. starkeyi* CBS 7786 grew comparatively well in hemicellulose hydrolysate and 30 g/L CG medium, but had lower growth compared to the tested red yeast strains in higher concentrations of CG.

### Testing of growth in a mixture of crude glycerol and hemicellulose

**Fig. 1:**
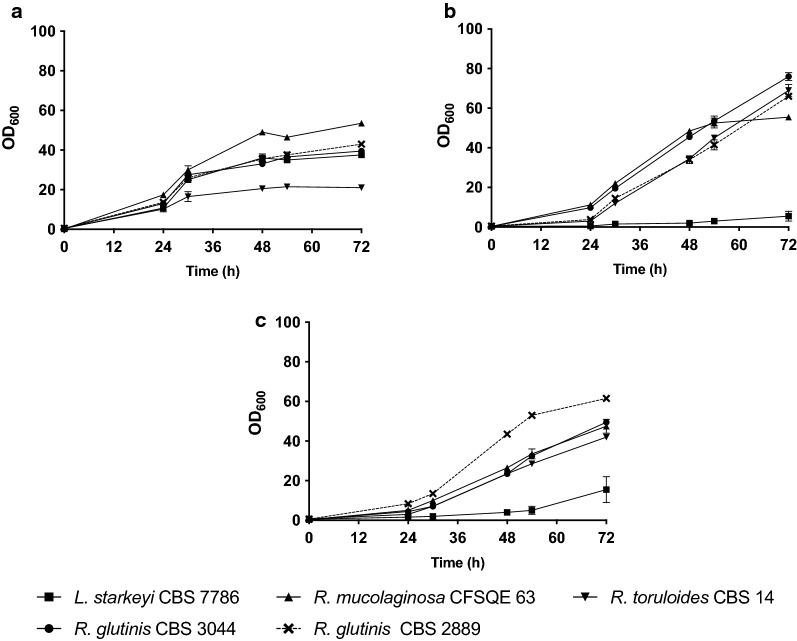
Growth of five strains tested in different media. **a** control medium, **b** hemicellulose hydrolysate mixed with crude glycerol and **c** crude glycerol + YNB media in shake flask cultures. 60 g/L Crude Glycerol, and 40% Hemicellulose hydrolysate. Strains were grown in duplicates

**Fig. 2:**
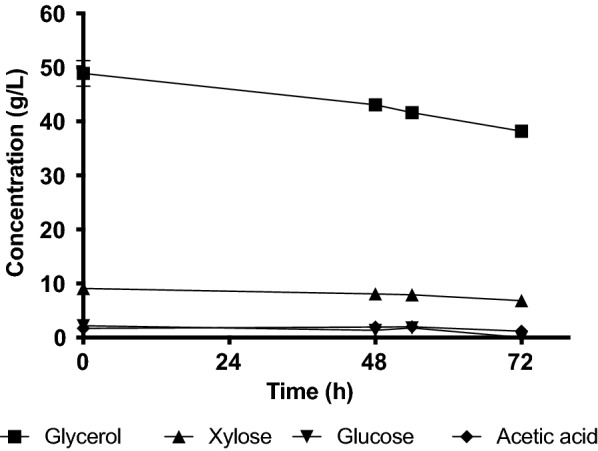
Carbon source consumption in shake flask cultivation of *L. starkeyi* CBS 7786 in sHH40CG60 medium. Xylose had a final concentration of 6.8 g/L; final concentration of glycerol was 38.2 g/L. The values were obtained from duplicate cultivations

**Fig. 3:**
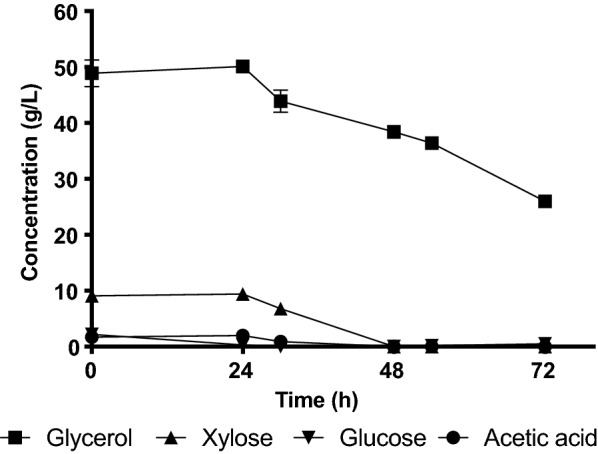
Carbon source consumption in shake flask culture of *R. toruloides* CBS 14 grown in sHH40CG60. Xylose was consumed after 30 h of cultivation, glycerol levels were dropping to a final concentration of 26.0 g/L after 70 h of cultivation. Cultivations were grown in duplicates

**Fig. 4:**
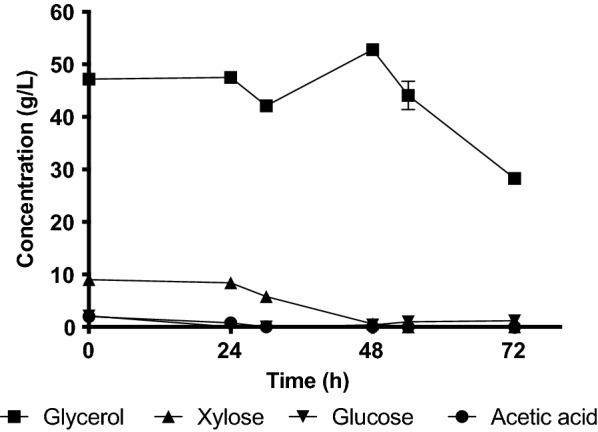
Carbon source consumption in shake flask culture of *R. glutinis* CBS 3044 grown in sHH40CG60. Xylose was consumed after 30 h of cultivation, glycerol levels dropped to a final concentration of 28.3 g/L after 70 h. Cultivations were grown in duplicates

To evaluate the effect of mixing CG and HH in the cultivation media we tested five strains, *L. starkeyi* CBS 7786, *R. toruloides* CBS 14, *R. glutinis* CBS 3044, *R. glutinis* CBS 2889 and *R. mucolaginosa* CFSQE 63, for their growth on these mixed substrates and compared it to cultivations with either glucose (positive control) or crude glycerol as sole carbon source (Figs. 1–4). The strains were chosen among those that showed good growth on crude glycerol and hemicellulose hydrolysate media. There were varying responses of the strains to the different carbon sources. In the glucose control medium (Control media), *R. toruloides* CBS 14 had the lowest OD_600_ after 96 h of cultivation (21), while *R. mucolaginosa* CFSQE 63 reached an OD_600_ more than twice as high as *R. toruloides* after 96 h*.* In mixed media (sHH40CG60 media), results were almost the opposite. *R. toruloides* had the second highest OD_600_ after *R. glutinis* CBS 3044*.* Out of the red yeasts, *R. mucolaginosa* CFSQE 63 had the lowest OD_600_. In media with crude glycerol (sCG60 media) only, *R. glutinis* CBS 2889 had the highest OD_600_ and *R. glutinis* CBS 3044 reached the second highest OD_600_. Interestingly, faster growth was observed for the red yeasts when cultivated using a mixed medium, compared to cultivations using pure CG.

*L. starkeyi* CBS 7786 showed slow growth on both CG alone and the mixture of CG and HH (Fig. 1). In contrast to the red yeasts, mixing CG with HH had no stimulating effect but was rather inhibiting the growth. In sHH40CG60 media, from starting glycerol concentration of approximately 50 g/L, *Lipomyces starkeyi* CBS 7786 consumed only around 25% of this, with 38.2 g/L glycerol still remaining after 72 h cultivation (Fig. 2). It was also only able to assimilate a small proportion of xylose, which was consumed by the other tested strains within 30 h.

In *R. toruloides* CBS 14 cultivations, the remaining glycerol concentration was 26 g/L after 70 h of cultivation. Xylose consumption started after 30 h (Fig. 3). *R. glutinis* CBS 3044 (Fig. 4), also consumed xylose between the 30 and 48-h measurement. The remaining concentration of glycerol was 28.3 g/L.

For *R. mucolaginosa* CFSQE 63 and *R. glutinis* CBS 2889 it turned out to be difficult to measure the concentration of carbon sources in the medium, because during filtration of all samples taken after inoculation, filter membranes rapidly got clogged. Although the supernatants were centrifuged before filtration as described before [19], it was in principle not possible to filter the supernatant and for the little amount of liquid that passed through the filter, results were not reproducible (results not shown). Production of exopolysaccharides has been shown in several strains of *Rhodotorula* spec. [29–31], and the clogging of the filters may be due to production of those polysaccharides in these two strains. Due to the difficulties in analysing the metabolites in the fermentations, we did not further investigate these strains in this study.

### Fermentation in bioreactors

Two of the investigated strains, *R. toruloides* CBS 14 and *R. glutinis* CBS 3044, were tested under controlled conditions in 500 ml fermenters for their abilities to utilise crude glycerol and hemicellulose hydrolysate and mixtures of them.

Cultivations were performed in 55 g/L of CG (CG55) as sole carbon source for the control and in a mixture of 55 g/l glycerol with 10% HH (HH10CG55). The C/N ratio of HH10CG55 mixture was 53.6, and CG50 C/N ratio was 48 which are slightly below the reported optimum for lipid production of 60–100 [32]. To obtain an impression on the lipid accumulation kinetics, the intracellular lipid content during fermentation was checked over the whole fermentation time using our recently established FT-NIR-based method for intracellular lipid determination [33].

**Fig. 5:**
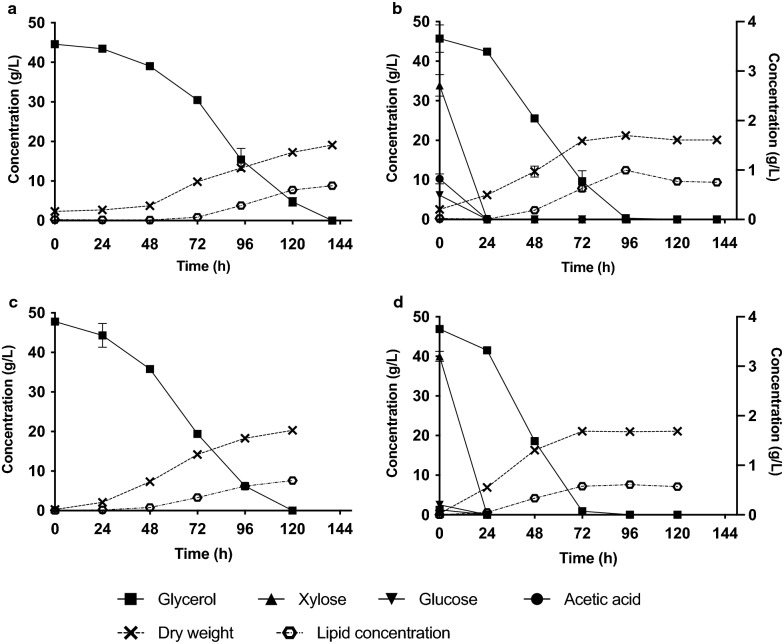
Dry weight, lipid concentration and carbon source consumption over time in media with CG as sole carbon source (CG55) or mixed carbon source (HH10CG55). **a** *R. toruloides* CBS 14 bioreactor cultivation CG55. **b** *R. toruloides* CBS 14 bioreactor cultivation in HH10CG55; **c** *R. glutinis* CBS 3044 bioreactor cultivation in CG55. **d** *R. glutinis* CBS 3044 bioreactor cultivation in HH10CG55; Glucose, xylose and acetic acid are shown on the right y axis. All cultures were done in triplicates

In the cultivation of *R. toruloides* CBS 14, glycerol was faster consumed in the HHCG mixture, compared to the CG control. After 72 h, more than 75% of all glycerol was consumed in the mixed medium, and after 96 h no glycerol was detectable any more. In the control with only CG as carbon source, even after 120 h of cultivation 4.7 g/L glycerol was left in the culture and complete consumption was only found after 144 h. Moreover, the highest cell dry weight (21.2 g/L) and lipid concentration (12.5 g/L) were reached in the HHGC mixture after 96 h cultivation (Fig. 5b). In the culture with CG as sole carbon source, the maximum dry weight (19.1 g/L) and lipid concentration (8.81 g/L) were detected after 140 h cultivation (Fig. 5a).

Similarly, an activating effect was observed for *R. glutinis* CBS 3044 when cultivated in HHGC compared to CG as sole carbon source (Fig. 5c, d). The glycerol was almost finished after 72 h, while in the control with only CG, more than 40% of the initial carbon source was present at this time. *R. glutinis* CBS 3044 in CG consumed glycerol slightly faster than *R toruloides* CBS 14 – all of it was consumed by 120 h. In the culture with CG as sole carbon source, the maximum dry weight (20.3 g/L) and lipid concentration (7.6 g/L) were detected after 120 h cultivation (Fig. 5c). Highest lipid concentration (7.6 g/L) was reached in the HHGC mixture after 96 h cultivation (Fig. 5d). The highest cell dry weight (21.0 g/L) was achieved after 72 h and it only changed by 0.1 g/L during the next 48 h.

In addition, we analysed the specific lipid production rates in 55 g/L CG media (Additional file 1: Fig. S1). The lipid accumulation was highest in both red yeast strains in media containing HH. *R. toruloides* had similar specific lipid production rates compared to *R. glutinis* (maximum specific rates of 0.014 vs 0.013 g/g/h)*,* but growth was delayed by 24 h. According to a fixed effect linear model the hemicellulose hydrolysate additive had a significant impact on *R. toruloides* lipid accumulation (*p* < 0.001) but not on *R. glutinis* (*p* = 0.0625). The volumetric production rates followed the same trend as the specific rates. *R. toruloides* CBS14 in mixed medium had its maximum production rate (0.23 g/L lipid per hour) between 48 and 72 h of cultivation, in CG medium *R. toruloides* CBS14 had the highest rate (0.16 g/L lipid per hour) between 96 and 120 h. *R. glutinis* CBS 3044 in mixed medium had its maximum production rate (0.15 g/L lipid per hour) between 24 and 48 h of cultivation, in CG medium *R. glutinis* CBS 3044 had the highest rate (0.12 g/L lipid per hour) between 72 and 96 h.

These results confirmed under controlled conditions the activating effect of mixing HH and CG, which we already observed for red yeasts in shake flasks. To further verify this effect, we performed a variety of further controlled fermentations with varying concentrations of CG and HH (Additional file 2: Fig. 2 and Additional file 3: Fig. 3). For all tested concentrations (with a C/N ratio from 38.3 to 72) we found a more rapid glycerol consumption and lipid formation in cultures with a mixed carbon source.

Lipid content, concentration, yield and biomass at the end of fermentation times are presented in Table 1. In most cases, the final lipid yields per g consumed carbon source were not significantly different between the cultivations with mixed carbon source and CG alone, the major effect was due to more rapid initial glycerol consumption. The highest lipid content was measured in *R. toruloides* CBS 14 grown in HH40CG60 medium (C/N ratio 72.2), which was probably due to the higher concentration of carbon source and higher C/N ratio. In addition, a comparatively high lipid yield (0.23 g lipids per g consumed carbon source) was observed. However, in this cultivation, not all glycerol was consumed and it turned out that the glycerol consumption rate decreased towards the end of the culture (Additional file 3: Fig. 3). In the cultivation with HH10CG55, all carbon source was consumed and the yield was even higher (0.25). The lipid yields of *R. glutinis* CBS 3044 were slightly lower compared to *R. toruloides* CBS 14.

**Table 1:** Endpoint lipid content, concentration, yield and specific lipid production in *Rhodotorula toruloides* CBS 14 and *Rhodotorula glutinis* CBS 3044 at the end of fermentation on different media

Strain	Media carbon source	Cell lipid content (%)	Max. lipid concentration (g/L)	Lipid	Final biomass (g/L)
				yield	
				(g lipid/ g carbon source)	
*R. toruloides* CBS 14	10% HH	46.8 ± 1.0	12.5 ± 0.5	0.25 ± 0.02	20.1 ± 0.7
	55 g/L CG				
	55 g/L CG	46.2 ± 5.6	8.8 ± 0.1	0.20 ± 0.01	19.1 ± 0.3
	10% HH	45.8 ± 3.5	6.5 ± 0.5	0.18 ± 0.02	13.5 ± 0.01
	50 g/L CG				
	50 g/L CG	46.3 ± 1.7	6.3 ± 0.2	0.19 ± 0.01	13.5 ± 0.01
	40% HH	54.6 ± 1.5*	10.6 ± 0.3*	0.23 ± 0.01*	19.4 ± 0.0*
	60 g/l CG				
	60 g/L CG	40.5 ± 2.7*	5.7 ± 0.4*	0.17 ± 0.00*	28.0 ± 0.5*
*R. glutinis* CBS 3044	10% HH	36.4 ± 0.6	7.6 ± 0.5	0.15 ± 0.01	20.3 ± 0.3
	55 g/L CG				
	55 g/L CG	37.3 ± 1.9	7.1 ± 0.1	0.16 ± 0.01	21.1 ± 0.01

Averages of triplicate cultivations with standard deviation, *average of duplicate cultivations with average deviation. lipid yield = $(\text{Total lipid concentration})/(\text{Total carbon consumed})$

### Fatty acid analysis

The average fatty acid composition for *R. toruloides* CBS 14 is presented in Table 2. In all cases, there were four dominant fatty acids; oleic acid, palmitic acid, linoleic acid and stearic acid. Oleic acid (C18:1) was the most common fatty acid with the highest proportion of 46.0% of total identified fatty acids. The second major compound was palmitic acid (C16:0) with its highest proportion of 28.0%. The third major fatty acid was linoleic acid (C18:2), the highest proportion of which was 16.3% of total identified fatty acids. The last major fatty acid was stearic acid (C18:0). Its highest proportion was 12.6% of total identified fatty acids. Proportions of the remaining fatty acids can be seen in Table 2. The difference in proportion of the fatty acid concentration in samples that were taken from same cultures at 96 h and 144 h was not larger than four percent. The biggest changes were oleic acid for palmitic acid in HH10CG50 media and the opposite for CG50 after 48 h. It should also be noted that behenic acid (C22:0) was above detectable levels only in cultivations using CG60 or HH10CG50, after 90 h cultivation.

**Table 2:** Average fatty acid composition of *R. toruloides* CBS 14 (*n* = 2) in different media (percent (%) of total identified ± mean absolute deviation)

Fatty acid	HH40CG60	CG60	HH10CG50	CG50
C14:0	1.1 ± 0.1	0.9 ± 0.1	1.0 ± 0.1	1.2 ± 0.0
C16:0	25.6 ± 0.0	22.2 ± 0.1	26.0 ± 0.4	28.0 ± 0.6
C16:1	0.8 ± 0.1	0.9 ± 0.1	0.9 ± 0.1	0.8 ± 0.0
C18:0	11.3 ± 0.3	12.6 ± 0.1	12.1 ± 1.5	12.2 ± 0.0
C18:1	45.6 ± 0.0	46.0 ± 0.1	40.7 ± 0.3	39.6 ± 0.5
C18:2	12.0 ± 0.0	12.1 ± 0.1	14.0 ± 1.2	13.8 ± 0.0
C18:3	2.1 ± 0.1	3.2 ± 0.1	2.9 ± 0.3	2.5 ± 0.0
C22:0	N/D	0.6 ± 0.0	0.5 ± 0.0	N/D
C24:0	0.6 ± 0.1	1.4 ± 0.1	1.4 ± 0.3	0.8 ± 0.0

## Discussion

Crude glycerol proved to be a problematic substrate for oleaginous yeasts. Only one third of all tested strains showed substantial growth in plate-cultivation tests with CG as sole carbon source, all except one being basidiomycetes. All positively tested yeast strains were able to grow in medium containing up to 120 g/L CG, although there were differences in growth rates between the strains. All strains showed comparatively good growth up to CG concentrations of 60 g/L, which is in accordance with results presented by Chatzifragou et al. [34].

Only one of the tested *Lipomyces* strains showed substantial growth on crude glycerol, but considerably slower than the tested red yeasts, in fact, its growth was already retarded at CG concentrations of 60 g/L. The reason for low tolerance of *Lipomyces* strains to crude glycerol in all cases is unknown as the literature clearly shows that they can utilise glycerol effectively [9]. Most likely some inhibitors from ash were the cause of low tolerance. Other inhibitors in trace amount like soaps, free fatty acids and residual catalysts might also be present in our samples due to differences in purification processes of each biodiesel production plant. Another observation was that mixing both CG and HH had a negative effect on both growth and assimilation of glycerol and xylose for *L. starkeyi*. The presence of xylose had a slightly negative effect on lipid accumulation in other oleaginous yeasts [35]. However, from the current data we cannot draw any valid conclusion about the physiological background of the different behaviour of the red yeasts and *L. starkeyi* in mixtures of CG and HH.

In contrast, for the two in detail tested red yeast strains, we found a considerable activating effect of mixing HH with CG. Cells started consuming glycerol and producing lipids much earlier than in culture without HH. At least for *R. toruloides* CBS 14 there was a clear effect on specific lipid production after the additional carbon sources were consumed. Thus, the higher glycerol conversion was not only due to an increased biomass because of the additional carbon sources but to an increased metabolic activity. The impact on specific lipid production was less pronounced in *R. glutinis* CBS 3044, still, the activating effect of adding HH was clearly visible for this strain as well.

From the results it can be concluded, that an addition of HH to the culture media has a positive effect on lipid production rate for *Rhodotorula* species. The addition of HH also had an improving effect on biomass production. An addition of just 10% of HH (approximately 2.6 g/L xylose, 0.6 g/L glucose, 0.8 g/L acetic acid) resulted in consumption of all available carbon sources within 96 h of cultivation. Cultures without HH needed at least 24 h more to consume all glycerol in the media. The addition of HH did not impact specific glycerol uptake significantly but it accelerated reaching maximum lipid production rate in *R. toruloides.* This increased rate of lipid production by supplementing crude glycerol media with hemicellulose hydrolysate has not been reported before for yeast, and this is worth further investigations. In previous studies mixed substrates of lignocellulosic hydrolysates supplemented with glycerol have been performed. Shen et al. found a similar positive effect in *Trichosporon fermentans* of faster glycerol metabolism when adding sweet potato waste, which contained 30 g/L of reducing sugars. Gong et al. described increased lipid yield and productivity in *Cutaneotrichosporon curvatum* (synonym *Cryptococcus curvatus*) when cultivating it in a mixture of corn stover hydrolysate and glycerol [10, 36]. However, in another study with the oleaginous zygomycete *Cunninghamella echinulata*, where tomato hydrolysate was used with glycerol no such effect has been observed [37]. Our results showed higher total lipid concentrations when compared to recently published studies, which investigated mixtures with xylose and crude glycerol of higher quality (90–95% vs our 80%) as growth substrates [35]. This may be due to better controlled conditions in bioreactors compared to shake flask cultures.

The 40% HH supplement to the culture media used to grow *R. toruloides* also resulted in the highest average lipid content. Even the 10% HH supplement apparently decreased the time in which lipid contents of > 40% were achieved. Without HH addition, lipid content in CG media (CG50 and CG60) was similar to that in experiments with other *R. toruloides* strains grown using glycerol as carbon source [38]. It is possible that with proper adjustment of the ratio between HH and CG an even higher lipid concentrations could be achieved in less than 96 h. Shortening the fermentation time has been described as a crucial aspect of an efficient process, as aerobic fermentations, in contrast to anaerobic processes, require substantial energy input for aeration [22]. The calculated yields of lipids per consumed substrate in *R. toruloides* in mixed media (0.18–0.25) were higher or comparable to previously reported ones using glucose, which were 0.21 g/g [39] or pure glycerol with *R. toruloides* 0.16 g/g [35]. In *C. curvatum* in cornstover hydrolysate, lipid yield was 0.18 g/g [36].

In our hands, it was necessary to add additional nitrogen to the hydrolysates when cultivating red yeasts, as the cells stopped growth and lipid accumulation during cultivation, probably due to too low nitrogen contents. For industrial processes, cheap and abundant nitrogen sources have to be identified. Food wastes are examples of such nitrogen sources, and recent results demonstrated that for instance waste milk or protein hydrolysates from chicken byproducts can be used for the production of yeast biomass [40, 41].

The activating effect by the addition of HH seems to be specific for red yeasts, as in *L. starkeyi* CBS 7786 the addition of HH had a rather inhibiting effect. The metabolism of xylose and glycerol are both generating glyceraldehyde-3-phosphate (G-3-P). In red yeasts, glycerol assimilation is possibly activated due to an increased amount of G-3-P. Glycerol is also required to form triacylglycerol (TAG), the major storage lipid in oleaginous yeasts. It is possible that the cell activates glycerol uptake to produce TAG from the additional carbon sources. Acetic acid could also play an important role in this process, as it is converted to acetyl-CoA, the precursor of fatty acid production [42]. Nevertheless, for the moment we can only speculate on the mechanism of the activation of the lipid metabolism by HH in *Rhodotorula* species. Further investigations are required to understand this phenomenon, for instance studies of the transcriptome of the yeasts.

The fatty acid profiles achieved for *R. toruloides* in this study are similar to those of vegetable oils like palm oil, and match biodiesel profile requirements [20, 43, 44]. Moreover, the yeast oil can also be used in other applications, such as for the replacement of vegetable oil in fish feed [24]. Interestingly, the fatty acid profile did not vary much despite that different cultivation media were used and the time of cultivations had varied.

## Conclusions

This study proved that the low-value side products CG and hemicellulose can be used for production of lipids. Addition of HH to CG considerably increased lipid production rate in *R. toruloides* and to some extent in *R. glutinis*. Based on the results of our study it seems to be possible to add value to side streams of biodiesel production and pulp and paper industry. Glycerol utilisation and shorter cultivation times are crucial factors to improve the efficiency of biodiesel production based on oleaginous yeasts [22]. In addition, this study has proven that *R. toruloides* CBS 14 has a good potential for microbial lipid production utilising CG and HH as carbon sources during cultivation.

## Materials and methods

### Yeast strains

Strains used are presented in Table 3.

**Table 3:** List of oleaginous yeast strains tested for the capability to utilise crude glycerol and hemicellulose hydrolysate as carbon source

Species	Strain
**Lipomyces**	
*lipofer*	CBS 944
*lipofer*	CBS 5842
*starkeyi*	CBS 1807
*starkeyi*	CBS 2512
*starkeyi*	CBS 6047
*starkeyi*	CBS 7536
*starkeyi*	CBS 7544
*starkeyi*	CBS 7545
*starkeyi*	CBS 7786
*starkeyi*	CBS 7851
*starkeyi*	CBS 7852
**Rhodotorula**	
*babjevae*	CBS 7808
*babjevae*	CBS 7809
*glutinis*	CBS 20
*glutinis*	CBS 2203
*glutinis*	CBS 2367
*glutinis*	CBS 2889
*glutinis*	CBS 2890
*glutinis*	CBS 3044
*glutinis*	CBS 5182
*glutinis*	CBS 5805
*glutinis*	CBS 7538
*glutinis*	CBS 9477
*graminis*	CBS 3043
*minuta*	CBS 8013
*mucolaginosa*	CFSQE 63
*toruloides*	CBS 14

CBS—Westerdijk Fungal Biodiversity Institute, Utrecht, The Netherlands, CFSQE—The Center for Food Safety and Quality Enhancement, Griffin, Georgia, USA

All strains were stored at − 80 °C in 50% v/v glycerol and pre-grown on YPD plates (glucose 20 g/L (≥ 99%, Fluka Analytical, France), yeast extract 10 g/L (Bacto™ Yeast Extract, BD, France), peptone 20 g/L (from casein, Merck KGaA, Germany), agar powder 20 g/L (VWR chemicals, Belgium, pH = 6,) in 4 °C and were re-streaked every 4 weeks.

All cultivation media were sterilised by autoclaving for 20 min at 121 °C and 4 bars unless stated otherwise. Fragile media components were sterile filtered using 0.2 µm syringe filters (Sarstedt, Germany). Hemicellulose hydrolysate was filtered using 0.45 µm bottle top unit filters followed by 0.2 µm bottle top unit filters (VWR, Belgium).

To prepare a pre-culture, three types of media were used: (a) YPD—Glucose 20 g/L (≥ 99%, Fluka Analytical, France), Yeast extract 10 g/L (Bacto™ Yeast Extract, BD, France), Peptone 20 g/L (from casein, Merck KGaA, Germany), pH = 6; (b) YPG—Glycerol 30 g/L, Yeast extract 10 g/L, Peptone 20 g/L, pH = 6; and (c) YPXG – Xylose 10 g/L, Glycerol 20 g/L, Yeast extract 10 g/L, Peptone 20 g/L, pH = 6.

All pre—cultures were started from yeast colonies grown on YPD plates and run in 500 mL baffled flasks in 100 mL of media, except the pre-cultures for the plate screening tests, which were done in 100 mL shake flasks with 10 mL of media. All starter cultures were incubated at 25 °C, for 72 h.

For preparation of inoculum, cultures were collected into sterile 50 mL falcon tubes and centrifuged at 3000×* g* for 5 min. The supernatant was discarded and the pellet washed with a solution of sterile filtered NaCl (9 g/L). This process was repeated once. After refilling with the NaCl solution, OD_600_ was measured to calculate inoculation volume to reach a starting OD_600_ of ~ 1.

Media used in growth tests contained—1.7 g/L Yeast Nitrogen Base (YNB) w/o amino acids and 2 g/L ammonium sulphate (DifcoTM, Becton–Dickinson and Company, USA), with different concentrations of crude glycerol, hemicellulose hydrolysate or combinations of both, together with 0.1 M Potassium phosphate buffer pH 6. Tests were performed in 100 mL Erlenmeyer flasks, incubated at 25 °C on a rotary shaker at 120 rpm. In all shake flask tests, OD_600_ measurement was used to quantify cell growth. All media used are presented in Table 4.

**Table 4:** Media used in this study; HH—hemicellulose hydrolysate, CG—Crude glycerol, s—shake flask culture

Name	Media components	
Control	20 g/L glucose, YNB 1.7 g/L, 2 g/L NH_4_SO_4_, 0.1 M Potassium phosphate buffer pH 6, YE 0.75 g/L	
sHemi	40% HH	+ YNB 1.7 g/L, 2 g/L NH_4_SO_4_, 0.1 M Potassium phosphate buffer pH 6, YE 0.75 g/L
sCG30	30 g/L CG	
sCG60	60 g/L CG	
sCG90	90 g/L CG	
sCG120	120 g/L CG	
sHH40CG60	40% HH + 60 g/L CG	
HH40CG60	40% HH + 60 g/L CG	+ YNB 1.7 g/L, 2 g/L NH_4_SO_4_, YE 0.75 g/L, 1 g/L MgCl_2_
HH10CG50	10% HH + 60 g/L CG	
CG50	50 g/L CG	
CG60	60 g/L CG	
HH10CG55	10% HH + 55 g/L CG	
CG55	55 g/L CG	

C/N ratios were determined as described in [36]. For yeast extract a C/N ratio of 3.6:1 was assumed as described in [45], which also was confirmed by own analyses (unpublished results).

Hemicellulose hydrolysate was based on wheat-straw subjected to acid-based steam explosion which was processed by the Department of Biochemical Process Engineering Luleå University of Technology, Sweden, as described previously [46]. According to HPLC-measurements, HH contained 26.2 g/L xylose, 7 g/L glucose, 6.6 g/L acetic acid and trace amounts of arabinose (< 0.5 g/L). The pH of the HH was set to 6 by addition of 5 M NaOH. In the experiments, the HH was diluted with water.

Crude glycerol was provided by Perstorp Holding AB. The composition was 80% glycerol, 5% ash, 15% water, 0.5% methanol and trace amounts of feedstock—vegetable oil.

## Growth experiments

### Crude glycerol and hemicellulose growth test

To test strains for the ability to grow on CG, plates with YNB and agar (16 g/L) were used without carbon source. The inoculation cultures were diluted to reach a density of ~ 0.5 McFarland and plated on the testing plates. Testing plates were (I) a CG drop was placed in the centre of the plate, (II) a sterile disk soaked in 50% CG was placed in the centre of the plate and (III) negative control without any carbon source. All the plates were incubated for 72 h in 25 °C.

For confirming the growth on mixed CG and HH, six liquid media were used: Control, sCG3, sCG6, sCG9, sCG12 and sHemi (Table 4). 20 ml of media was used in 100 ml Erlenmeyer flasks. Cultures were grown for 96 h at 25 °C on a rotary shaker at 120 rpm, with OD_600_ measurements after 72 h and 96 h.

A mixture of CG and HH (sHH40CG60, C/N ratio ~ 78) was compared to CG alone (sCG60, C/N ratio ~ 58) or a control (Table 4). All samples were set up in duplicates. Cultures were grown for 72 h with OD_600_ measurements each 24 h and subsequently every 6 h.

### Bioreactor cultivations

Fermentations were performed in 500 mL bioreactors Multifors 2 (Infors HT, Switzerland) containing 350 ml medium. The fermentation conditions were 25 °C, pH = 6 (acid 3 M H_3_PO_4,_ base 5 M NaOH), stirrer starting at 200 rpm with maximum speed 800 rpm, aeration 0.3 L/min (0.9 vvm) and DOT set to 20%. Polypropylene glycol 2000 was added as antifoaming agent, when needed. Unless stated otherwise, each tested strain was cultivated in triplicate. The media tested are stated in Table 4. Fermentations were performed for 120 or 144 h depending on strain and carbon consumption. Biomass concentrations were determined by cell dry weight determination.

Specific lipid production was calculated for the midpoint of an according measuring interval:

$$Specific \, lipid \, production \,= \,(\Delta \, Lipid \, concentration )/(Average \, X*\Delta t)$$

$\Delta$ Lipids were determined as difference between the measured lipid concentration values after the respective midpoints and before. Average X were calculated from the mean of biomasses determined after and before the respective midpoints. $\Delta t$ was the time interval between the two measuring points.

### Analytical methods

The optical density (OD_600_) was measured at a wavelength of λ = 600 nm using CO8000 Cell density meter (Nordic Biolabs). HPLC and cell dry weight measurements were performed as described before [19]. To prepare samples for lipid extraction, harvested cells were freeze dried in vacuum for 72 h with condenser set to − 100 °C (CoolSafe Scanvac, LaboGene ApS, Denmark). Lipid concentration and fatty acid profile were determined as described earlier [19]. For rapid determination of lipid content we used our recently described FT-NIR method [33].

## Supplementary Information

**Additional file 1. Figure 1.** Specific lipid production rates of* R. toruloides* CBS 14 and R. glutinis CBS 3044 in 55 g/L crude glycerol media and 55 g/L crude glycerol media with 10% hemicellulose hydrolysate. Negative values are due to a decrase of lipid concentrations during the measuring interval.

**Additional file 2. Figure 2.*** R. toruloides* CBS 14, HH40CG60 media grown in duplicates, dry weight and change of compounds concentration in media over time, average lipid concentration was 10.57 g/L after 70h. Glucose, xylose and acetic acid are presented on secondary y axis.

**Additional file 3. Figure 3.** Bioreactor cultivation of* R. toruloides* CBS 14 in triplicates, (A) CG50 medium, (B) HH10CG50 medium. Glucose, xylose and acetic acid are presented on secondary Y axis.

## Data Availability

The datasets used and/or analysed during the current study are available from the corresponding author on reasonable request.

## Abbreviations

CG: Crude Glycerol
FAME: Fatty Acid Methyl Esters
FT-NIR: Fourier Transform Near-Infra-red
HH: Hemicellulose Hydrolysate
YNB: Yeast Nitrogen Base
YM: Yeast Extract–Malt Extract
YPD: Yeast Peptone Dextrose
HIP: Hexane: Isopropanol
