# Supplementary figures and images for: Microbial lipid production from crude glycerol and hemicellulosic hydrolysate with oleaginous yeasts
Source: Biotechnol Biofuels. 2021 Mar 12;14:65. doi: 10.1186/s13068-021-01916-y (PMC7953724; doi:10.1186/s13068-021-01916-y)

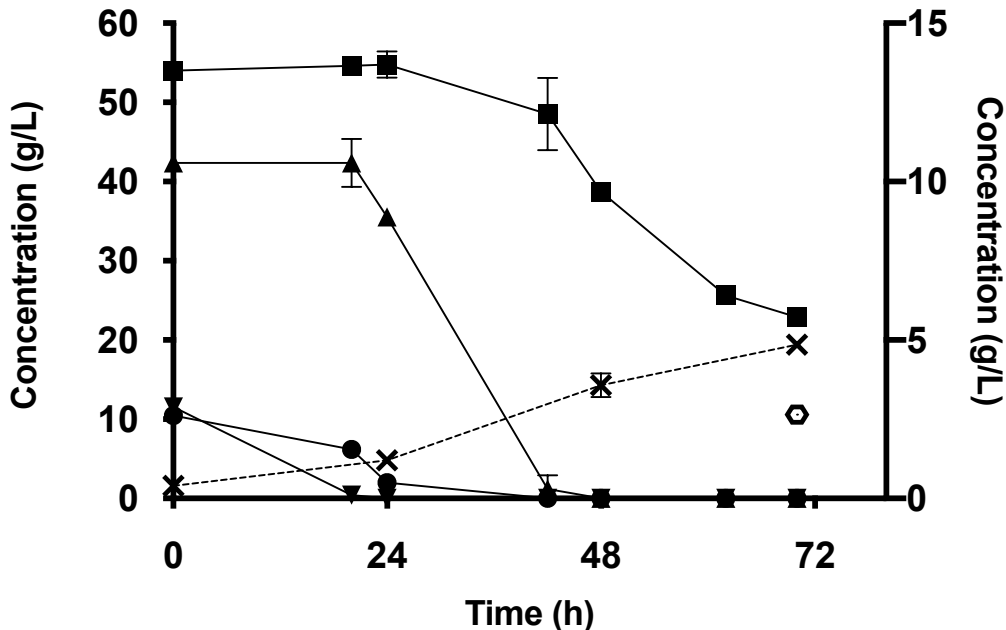

■ Glycerol    ▲ Xylose    ▼ Glucose    ● Acetic acid  
○ Lipid concentration    × Dry weight

Supplement: Supplementary file 1 — Additional file 1. Figure 1. Specific lipid production rates of R. toruloides CBS 14 and R. glutinis CBS 3044 in 55 g/L crude glycerol media and 55 g/L crude glycerol media with 10% hemicellulose hydrolysate. Negative values are due to a decrase of lipid concentrations during the measuring interval. [file 13068_2021_1916_MOESM1_ESM.pdf]

A

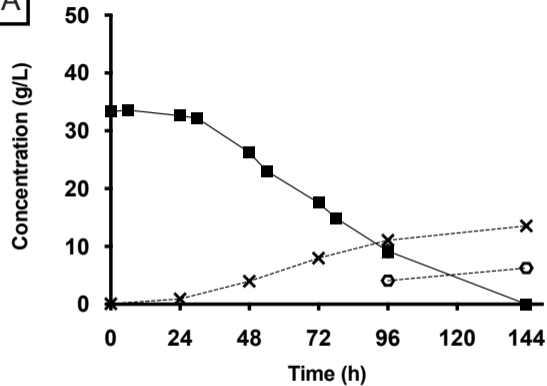

■ Glycerol

-X- Dry weight

▲ Xylose

-○- Lipid concentration

B

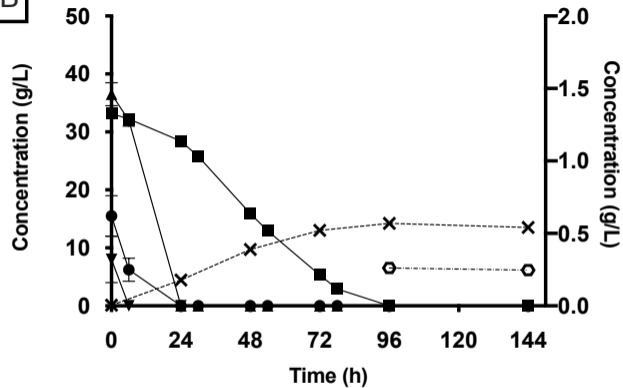

● Acetic acid

Supplement: Supplementary file 2 — Additional file 2. Figure 2. R. toruloides CBS 14, HH40CG60 media grown in duplicates, dry weight and change of compounds concentration in media over time, average lipid concentration was 10.57 g/L after 70h. Glucose, xylose and acetic acid are presented on secondary y axis. [file 13068_2021_1916_MOESM2_ESM.pdf]
