# Supplementary material for: Microbial lipid production from crude glycerol and hemicellulosic hydrolysate with oleaginous yeasts
Source: Biotechnol Biofuels. 2021 Mar 12;14:65. doi: 10.1186/s13068-021-01916-y (PMC7953724; doi:10.1186/s13068-021-01916-y)
Supplement: Supplementary file 3 — Additional file 3. Figure 3. Bioreactor cultivation of R. toruloides CBS 14 in triplicates, (A) CG50 medium, (B) HH10CG50 medium. Glucose, xylose and acetic acid are presented on secondary Y axis. [file 13068_2021_1916_MOESM3_ESM.pdf]

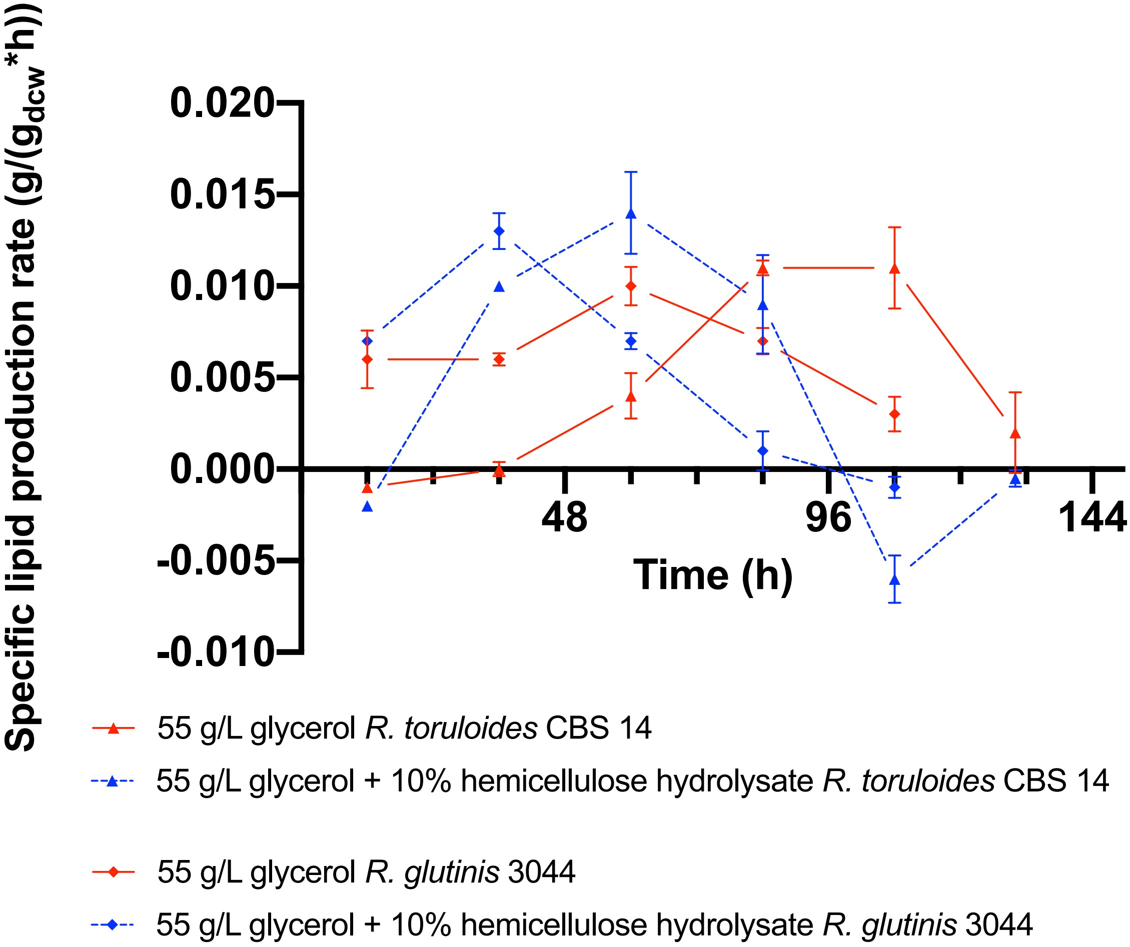

Specific lipid production rate (g/(g$_{dcw}$*h))
0.020
0.015
0.010
0.005
0.000
-0.005
-0.010
48
96
144
Time (h)
55 g/L glycerol *R. toruloides* CBS 14
55 g/L glycerol + 10% hemicellulose hydrolysate *R. toruloides* CBS 14
55 g/L glycerol *R. glutinis* 3044
55 g/L glycerol + 10% hemicellulose hydrolysate *R. glutinis* 3044
